# Magnetic resonance guided focused ultrasound surgery (MRgFUS) treatment of osteoid osteoma: a prospective development study

**DOI:** 10.1186/2050-5736-3-S1-O44

**Published:** 2015-06-30

**Authors:** Alessandro Napoli, Fulvio Zaccagna, Gaia Cartocci, Brachetti Giulia, Gianluca Caliolo, Fabrizio Andrani, Carlo Catalano

**Affiliations:** 1 University of Rome – Sapienza, Rome, Italy

## Background/introduction

To investigate mid- to long-term efficacy of MRgFUS in the treatment of symptomatic osteoid osteomas.

## Methods

This prospective, IRB approved study involved 29 consecutive patients with clinical and imaging diagnosis of Osteoid Osteoma; all patients underwent MRgFUS ablation (ExAblate, InSightec; Discovery 750 MR unit, GE). Lesions located in vertebral body were excluded; prior RFA or surgery was not considered an exclusion criteria. Patients received therapy using MRgFUS, delivered toward the nidus, identified on MRI and/or CT. Primary endpoints were adverse events (serious and otherwise) and pain relief assessed using questionnaires on Visual Analog Pain Score (VAS) and daily intake of Non-steroidal drugs (NSAIDs). Patient’s follow-up, including clinical and imaging examinations, was established at 1, 12 and 24 months. As secondary endpoint, imaging examinations (CT and CE-MRI; Gd-BOPTA, Bracco) were used to evaluate inflammatory status after treatment and bone remodeling.

## Results and conclusions

29 patients (female 8; male, 21; mean age 23) were recruited for MRgFUS treatment; all safely completed the procedure. The treatment was well tolerated by all patients and no adverse events were recorded after and during 12-24 months follow-up period. A mean number of 4 ± 1.8 sonications with mean energy of 894 ± 209 J was necessary to complete the treatment. Three patients underwent staged treatment (1 post-RFA, 1 post surgery, 1 intrarticular position). Complete clinical response was found in 27/29 (93% CI 6–18) patients in term of pain absence and no intake of NSAIDs. There was a statistically significant difference (p=0,001) between baseline (7,9 ± 1,4) and follow-up values (0,7 ± 0,1) for pain severity, according to VAS. Two patients (0.6%) reported pain recurrence requiring both RFA. Imaging evaluation with CE-MRI demonstrated edema and hyperemia decrease in every lesion associated with complete response. At CT, bone remodeling was evident in all complete responders (27/29); in 15/27 (55%) patients, nidus fading was demonstrated. MRgFUS can be safely and effectively adopted for the treatment of Osteoid Osteoma. This application is totally non-invasive, carried out in a single session and with pain relief attainable since the following day after treatment. Our results also indicated a positive trend to bone restoration.

